# Expression profiles of switch-like genes accurately classify tissue and infectious disease phenotypes in model-based classification

**DOI:** 10.1186/1471-2105-9-486

**Published:** 2008-11-17

**Authors:** Michael Gormley, Aydin Tozeren

**Affiliations:** 1 School of Biomedical Engineering, Drexel University, Philadelphia, PA, USA

## Abstract

**Background:**

Large-scale compilation of gene expression microarray datasets across diverse biological phenotypes provided a means of gathering a priori knowledge in the form of identification and annotation of bimodal genes in the human and mouse genomes. These switch-like genes consist of 15% of known human genes, and are enriched with genes coding for extracellular and membrane proteins. It is of interest to determine the prediction potential of bimodal genes for class discovery in large-scale datasets.

**Results:**

Use of a model-based clustering algorithm accurately classified more than 400 microarray samples into 19 different tissue types on the basis of bimodal gene expression. Bimodal expression patterns were also highly effective in differentiating between infectious diseases in model-based clustering of microarray data. Supervised classification with feature selection restricted to switch-like genes also recognized tissue specific and infectious disease specific signatures in independent test datasets reserved for validation. Determination of "on" and "off" states of switch-like genes in various tissues and diseases allowed for the identification of activated/deactivated pathways. Activated switch-like genes in neural, skeletal muscle and cardiac muscle tissue tend to have tissue-specific roles. A majority of activated genes in infectious disease are involved in processes related to the immune response.

**Conclusion:**

Switch-like bimodal gene sets capture genome-wide signatures from microarray data in health and infectious disease. A subset of bimodal genes coding for extracellular and membrane proteins are associated with tissue specificity, indicating a potential role for them as biomarkers provided that expression is altered in the onset of disease. Furthermore, we provide evidence that bimodal genes are involved in temporally and spatially active mechanisms including tissue-specific functions and response of the immune system to invading pathogens.

## Background

Gene expression is controlled over a wide range at the transcript level through complex interplay between epigenetic modifications, DNA regulatory proteins, and microRNA molecules [1-3]. Genome-wide screening of expression profiles has provided an expansive perspective on gene regulation in health and disease. For example, identification of constitutively expressed housekeeping genes has aided in the inference of sets of minimal processes required for basic cellular function [4,5]. Similarly, we have identified and annotated genes with switch-like expression profiles in the mouse and human, using large microarray datasets of healthy tissue [6]. Genes with switch-like expression profiles represent fifteen percent of the human gene population. Classification of samples on the basis of bimodal or switch-like gene expression may give insight into temporally and spatially active mechanisms that contribute to phenotypic diversity. Given the variable expression of switch-like genes, they may also provide a viable candidate gene set for the detection of clinically relevant expression signatures in a feature space with reduced dimensionality.

The high-dimensionality inherent in genome-wide quantification makes extracting meaningful biological information from gene expression datasets a difficult task. Early attempts at genome-wide expression analysis used unsupervised clustering methods to identify groups of genes or conditions with similar expression profiles [7-9]. Biological insight can be derived from the observation that functionally related or co-regulated genes often cluster together. Supervised classification methods require datasets in which the class of the samples is known in advance. Statistical hypothesis testing [10,11] is used to identify groups of genes that exhibit changes in expression associated with class distinction. Significant genes can be used to build decision rules to predict the class of unseen samples [12-14]. Unsupervised classification is better suited for class discovery whereas supervised classification is tailored for class prediction. In both of these complimentary approaches, dimension reduction can lead to increased classification accuracy.

Many simple unsupervised learning algorithms rely on distance metrics to either partition profiles into distinct groups [15,16] or build clusters from pair-wise distances in a nested, hierarchical fashion [9]. The optimal number of clusters must be defined heuristically or in advance and confidence in cluster membership is difficult to determine. Model-based clustering provides the necessary statistical framework to address these concerns while allowing for class discovery. In model-based clustering, it is assumed that similar expression profiles are generated as draws from a set of multivariate Gaussian random variables. Clusters are identified by fitting the parameters of the cluster-specific distributions to the data. Expectation-maximization [17-19] or Bayesian methods [20-22] are used for optimization. Estimation of the number of clusters as well as the incorporation of confidence in cluster membership is implicit in this process.

Methods such as unsupervised, supervised and model-based classification provide the means to evaluate switch-like gene expression patterns in high-dimensional datasets profiling diverse biological conditions. For this purpose, we compiled two large-scale gene expression microarray datasets from publicly available data repositories. The first dataset included samples spanning nineteen different tissue types from healthy donors. The second dataset included samples from donors with one of a number of infectious diseases including HIV-1 infection, hepatitis C, influenza, and malaria. Our results demonstrate that switch-like genes exhibit tissue and disease-specific expression signatures. Dimension reduction of genome-wide expression data through the identification of switch-like genes enabled highly accurate classification of samples into tissue-specific and disease-specific clusters. Moreover, analysis of activated switch-like genes in various disease and tissue types revealed that these genes participate in specialized or temporally active mechanisms. Further study of genes in the switch-like gene set may provide biologically significant information about the molecular basis of phenotype distinction.

## Results

### Three hundred bimodal genes classify nineteen tissue types with high accuracy in model-based classification

**Figure 1:**
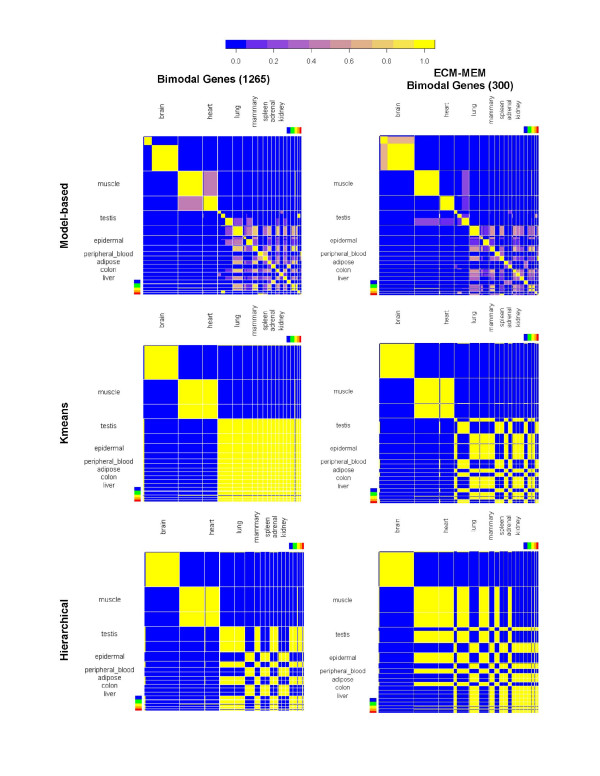
**Model-based clustering of bimodal gene expression identifies cohesive clusters in 19 tissue types**. Heat map representation of posterior pairwise probabilities for classification of tissue phenotype. Left column: classification with 1265 bimodal genes. Right column: classification with 300 bimodal genes translated into extracellular matrix or plasma membrane proteins. Top row: Model-based clustering, identifies all tissues distinctly. Middle and bottom rows: Kmeans and hierarchical clustering classify samples into three/four tissue types: brain, cardiac and skeletal muscle and remaining tissues. Blue, green, yellow, orange and red regions of color bar indicate ovary, stomach, small intestine, pancreas and thymus tissue samples respectively. Tissues in the heat map were ordered according to decreasing sample size from left to right.

A model-based classification algorithm [23] partitioned a set of 407 microarray samples into bins specific to 19 different tissue types (Figure 1). Classification was based either on the expression of the complete list of 1265 human switch-like genes (Figure 1 Column 1) or a subset of this list containing 300 bimodal genes translated into extracellular matrix or plasma membrane proteins (Figure 1 Column 2). Additional file 1 lists the Affymetrix probe set identifiers of the bimodal genes along with the full gene name and the dominant mode ("on" vs. "off" or "high" vs. "low") of expression in four tissues (brain, skeletal muscle, cardiac muscle and lung tissue). Heat maps shown in Figure 1 depict the posterior pairwise probability matrix for each pair of samples. The color of square elements of the heat maps indicate the number of partitions in which two samples are assigned to the same cluster, with yellow being the maximum and blue the minimum. Rows and columns of the heat map are organized to group samples of the same tissue type together. The figure shows that model-based classification correctly grouped microarray samples into tissue-specific clusters, even for tissues with as few as five microarray samples. Two distance-based clustering algorithms, Kmeans and hierarchical clustering, identified brain-specific (89 samples) and skeletal/cardiac muscle-specific clusters (64/38 samples, respectively) but failed to differentiate between tissues with smaller number of samples (Figure 1, Table 1). Consistent with the heat maps shown in Figure 1, the Adjusted Rand Index (ARI) values shown in Table 2 shows that model-based clustering outperformed distance-based algorithms in unsupervised classification of tissue phenotypes. Our results indicate that a set of 300 bimodal genes whose products localize to the cell membrane or extracellular matrix compartments are determinants of tissue type for the nineteen tissues listed in Table 1. Cell-cell/ECM interactions activate downstream transcriptional programs that regulate a diverse set of processes including growth, proliferation, apoptosis, and cell motility [24,25] and have often been associated with pathogenesis in muscular dystrophy, multiple sclerosis, and various cancers [26-29]. Noting that the tissue-specific sample size in the microarray data ranged from 5 to 89 (Table 1), results with model-based classification indicate the strength of tissue-specific signatures in global gene expression and the ability of bimodal genes to capture such signatures. Results also indicate that a subset of bimodal genes whose products are positioned either in the extracellular matrix or cell membrane is sufficient to identify tissue-specificity in microarray data. Given the importance of ECM and MEM proteins in the regulation of cellular function, products of these genes may serve as candidate biomarkers or therapeutic targets in tissue-specific diseases.

**Table 1:** Microarray datasets used in this study

Tissue Phenotype Data		
Tissue	No. of Samples	Gene Expression Omnibus/Array Express Accn. #

Adipose	10	GSE3526

Adrenal	20	GSE3526, GSE8514, GSE2316

Brain	89	GSE3526, GSE7621, GSE7307, GSE2361, E_AFMX-11, E-TABM-20,

Colon	10	E-TABM-176, GSE8671, GSE9254, GSE9452

Epidermal	25	GSE1133, GSE2361, GSE3419, GSE3526, GSE7307

Heart	38	E_AFMX-11, E-MIMR-27, GSE1133, GSE2240, GSE2361, GSE3526, GSE3585, GSE7307

Kidney	10	E_AFMX-11, GSE2004, GSE2361, GSE3526, GSE7392

Liver	10	E_AFMX-11, GSE2004, GSE3526, GSE6764

Lung	26	E-MEXP-231, GSE10072, GSE1133, GSE2361, GSE3526

Mammary	15	E-TABM-66, GSE2361, GSE3526, GSE7307, GSE7904

Muscle	64	GSE10760, GSE2328, GSE3526, GSE5110, GSE6798, GSE7307, GSE9103,

Ovary	10	GSE2361, GSE3526, GSE6008, GSE7307

Pancreas	6	GSE1133, GSE2361, GSE7307

Peripheral blood	12	GSE7462, GSE8608, GSE8668, GSE8762, GSE9692

Small intestine	7	GSE2361, GSE7307

Spleen	12	GSE2004, GSE2361, GSE3526, GSE7307

Stomach	10	GSE2361, GSE3526, GSE7307

Testis	38	E_AFMX-11, GSE1133, GSE2361, GSE3218, GSE3526, GSE7307, GSE7808

Thymus	5	GSE1133, GSE2361, GSE7307

Infectious Disease		

Disease	No. of Samples	Gene Expression Omnibus/Array Express Accn. #

Hepititis C	147	GSE11190, GSE7123

HIV	41	GSE6740, GSE9927

Influenza A	28	GSE6269

Malaria	15	GSE5418

**Table 2:** Adjusted Rand Index compares observed partitions with true classification of samples in tissue phenotype data

	Kmeans	Hierarchical	Model-based
All bimodal genes	0.291	0.463	0.683
ECM/MEM genes	0.456	0.304	0.881

### Enrichment analysis reveals tissue-specific functions of "on" genes in brain, skeletal muscle, cardiac muscle, and lung tissue

**Figure 2:**
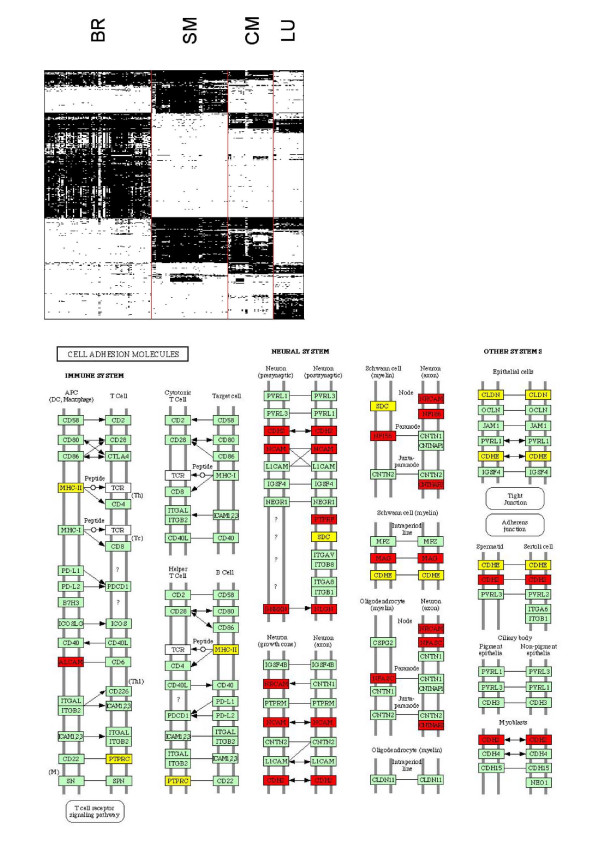
**Binarized expression of bimodal genes in brain, lung, skeletal muscle and cardiac muscle**. Top figure: heat map of 1265 bimodal gene expression in 217 tissue samples. A black/white point at *i*, *j *indicates gene *i *is "on"/"off" in sample *j*. Bottom figure: bimodal gene expression in KEGG cell adhesion molecules diagram. Genes marked with red are "on" in brain tissue and "off" in muscle tissue. Genes marked with yellow are "off" in muscle tissue.

Binomial tests were used to identify sets of bimodal genes biased toward the "on" mode in the tissues that are well-represented in our microarray dataset (> 25 samples). A gene by sample heat map (Figure 2A) shows the on-off modes of expression for all 1265 bimodal genes in 217 samples of brain, skeletal muscle, cardiac muscle and lung tissue. A black/white element of the heat map indicates a gene expressed in the "on"/"off" mode in a sample. Figure 2A shows that distinct clusters of "on" and "off" genes are observed in each of the four tissue types under consideration. We identified 542, 429, 322, and 278 genes over-represented in the "on" mode and 645, 778, 830 and 896 genes over-represented in the "off" mode in brain, skeletal muscle, cardiac muscle and lung tissue respectively. Overall, this figure indicates the abundance of switch genes with altered states in different tissues, resulting in accurate classification of tissue types using microarray data.

Functional enrichment analysis identified gene sets related to tissue-specific function in sets of bimodal genes expressed in the "on" mode in brain, skeletal muscle, cardiac muscle and lung tissues. The GO categories that are significantly enriched with bimodal genes that are "on" in brain tissue samples included neural tissue-specific processes including neural migration, adhesion, recognition and differentiation, nervous system development, and synaptic transmission (Table 3). Similarly, the list of enriched GO terms associated with skeletal and cardiac muscle tissue samples included terms related to muscle development and organization, muscle contraction, calcium ion binding, cellular metabolism and muscle-specific structures such as the sarcoplasmic reticulum, myofibril, sarcomere and z disc. A number of KEGG pathways are also enriched. The KEGG diagram summarizing cell adhesion molecules is enriched with genes turned "on" in brain tissue and genes turned "off" in muscle tissue (Figure 2B). Several of these cell adhesion molecules, such as CDH2, NCAM, NRXN, and NLGN, are expressed at synaptic junctions [30]. Another subset, including NFASC and CNTNAP2, is integral to the formation of myelinated neurons [31]. These results indicate that genes with bimodal expression patterns in the human genome tend to be involved with essential functions and structures in major tissues such as cardiac and skeletal muscle and brain.

**Table 3:** GO categories significantly enriched with "on" genes in brain tissue

**Biological Process**	**Cellular Component**	**Molecular Function**
▪ Neuron migration	▪ Cytoskeleton	▪ Actin binding
▪ Transport	▪ Microtubule	▪ GTPase activity
▪ Ion transport	▪ Microtubule associated complex	▪ Transmembrane receptor protein tyrosine
▪ Negative regulation of microtubule depolymerization	▪ Neurofilament	▪ Structural molecule activity
▪ Cell adhesion	▪ Membrane	▪ Strucutural constituent of cytoskeleton
▪ Neuron adhesion	▪ Integral to membrane	▪ Ion channel activity
▪ Transmembrane receptor protein tyrosine phosphatase signaling pathway	▪ Synaptosome	▪ Structural constituent of myelin sheath
▪ Synaptic transmission	▪ Cell junction	
▪ Neuromuscular synaptic transmission	▪ Axon	
▪ Nervous system development	▪ Growth cone	
▪ Synaptogenesis	▪ Synapse	
▪ Central nervous system development	▪ Postsynaptic membrane	
▪ Neuron recognition		
▪ Anterograde axon cargo transport		
▪ Neuron differentiation		

P-values < = 0.001 indicates significance.

### Model-based classification of infectious disease and immune response signature

**Figure 3:**
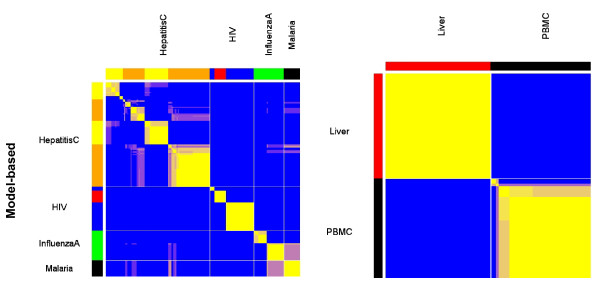
**Model-based clustering of bimodal gene expression classifies infectious disease states separately and identifies tissue-specificity in hepatitis C infection**. Heat map representation of pairwise posterior probabilities derived from model-based clustering of infectious disease expression data. Left column: Classification of hepatitis C, HIV, influenza A, and malaria profiled in peripheral blood mononuclear cells (PBMCs). Right column: Classification of hepatitis C infection profiled in peripheral blood mononuclear cells and liver biopsies.

Model-based clustering of bimodal gene expression led to accurate classification of disease phenotypes in an independent dataset of 221 microarray tissue samples profiling infectious diseases. Note that only normal tissue microarray data and not infectious disease data was used in the original annotation of switch-like genes. The posterior pairwise probability matrix derived from model-based clustering partitioned expression profiles of peripheral blood mononuclear cells (PBMC) into disease-specific clusters for HIV-1 infection, hepatitis C, influenza, and malaria (Figure 3). We focused on microarray data on PBMCs because these cells recognize pathogen-specific molecules in the circulation and lymphatic system and initiate the immune response [32]. In turn, pathogen recognition induces transcriptional activation of several host defense signaling pathways [33]. Results presented here indicate the potential of switch-like genes in the classification of disease states using microarray data. Furthermore, the use of switch genes along with model-based clustering leads to accurate classification of microarray data belonging to different tissue types that are infected by the same virus. Model-based clustering differentiated between samples of hepatitis C infection in PBMCs and liver biopsies (Figure 3). Thus, model-based clustering captures infectious disease signatures in microarray data in a tissue-specific manner.

**Figure 4:**
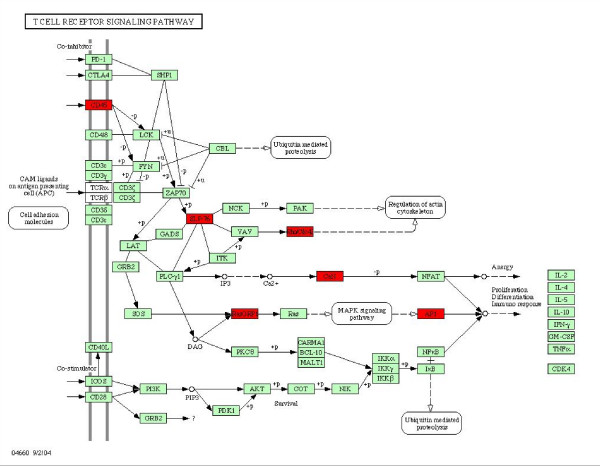
**Bimodal genes that were switched "on" as a result of HIV infection in KEGG T-cell receptor signalling pathways**. Bimodal genes marked with red are "on" in the KEGG T-cell receptor signaling pathway in HIV infection.

Next, we examined the switch states of bimodal genes in infectious disease associated microarray data. Of the 1295 bimodal genes analyzed, 192, 160, 148 and 117 genes were expressed in the "on" mode in the majority of samples from PBMCs in hepatitis C, influenza A, malaria, and HIV-1 infection, respectively. In liver biopsies from hepatitis C infected individuals, 301 bimodal genes are over-represented in the "on" mode. Biological processes commonly enriched in the set of bimodal genes expressed in the "on" mode in these diseases include B cell receptor signalling and humoral immune response involving circulating immunoglobulins (Table 4), processes that are central in the activation of the antigen-mediated, adaptive immune system [34-38]. Gene Ontology enrichment analysis for switch-like genes turned "on" in HIV-1 infection indicated significant enrichment of the biological processes of DNA methylation, translational initiation, negative regulation of protein kinase activity, and response to calcium (Table 4). The T-cell signaling pathway was also significantly enriched with bimodal genes expressed in the "on" mode in HIV-1 infection (Figure 4). The bimodal genes in this pathway code for the membrane receptor CD45 [39], kinase activator SLP-76 [40], RAS proteins RASGRP1 and Rho Cdc42, calcium binding protein CaN, and the transcription factor AP1 [41](Figure 4), all known to be crucial in immune defense system against viruses. Taken together, our results suggest a significant role for a subset of bimodal genes in the host-response to pathogens.

**Table 4:** GO categories significantly enriched with "on" genes in infectious disease

**Biological Process**	**Cellular Component**	**Molecular Function**
▪ Immune response^1, 2, 3, 4, 5^	▪ B cell receptor complex^1, 2, 4, 5^	▪ Antigen binding^1, 2, 4, 5^
▪ Humoral immune response by circulating immunoglobin^1, 2, 4, 5^	▪ Immunoglobulin complex, circulating^1, 2, 4, 5^	▪ Succinate dehydrogenase activity^2,3,4^
▪ Positive regulation of B cell proliferation^1, 2, 4, 5^	▪ Perinuclear region of cytoplasm^1, 2, 4, 5^	▪ RNA binding^3^
▪ Early endosome to late endosome transport^1, 2, 4, 5^	▪ External side of plasma membrane^1,4^	▪ Structural constituent of cytoskeleton^3^
▪ Positive regulation of peptidyl-tyrosine phosphorylation^1, 2, 4, 5^	▪ Membrane fraction^4,5^	▪ Protein binding^3^
▪ B cell receptor signaling pathway^1, 2, 4, 5^	▪ Cytoplasm^3,5^	▪ Electron-transferring-flavoprotein
▪ Activation of MAPK activity^1, 2, 4^	▪ Cytoskeleton^3^	▪ dehydrogenase activity^5^
▪ tRNA aminoacylation for protein translation^1,4^	▪ Actin cytoskeleton^3^	▪ Endopeptidase inhibitor activity^5^
▪ Antigen processing and Presentation^1,4^	▪ Extracellular region^5^	▪ Structural molecule activity^5^
▪ DNA methylation^3^	▪ Proteinaceous extracellular matrix^5^	▪ Extracellular matrix structural constituent^5^
▪ Translational initiation^3^	▪ Collagen^5^	
▪ Negative regulation of protein kinase activity^3^		
▪ Defense response^3^		
▪ Inflammatory response^4^		
▪ Hemocyte development^4^		
▪ Cell-cell adhesion^4^		
▪ Pyridine nucleotide biosynthetic process^4^		
▪ Respiratory burst^4^		
▪ Response to calcium ion^3,4^		
▪ Tricarboxylic acid cycle^5^		
▪ Cell adhesion^5^		
▪ Blood coagulation^5^		
▪ Sensory perception of sound^3,5^		

P-values < = 0.001 indicate significance in malaria, influenza A, hepatitis C-PBMCs and hepatitis C-Liver. P-values < = 0.01 indicate significance in HIV. ^1^malaria, ^2^influenza A, ^3^HIV, ^4^hepatitis C-PBMC, ^5^hepatitis C-liver

### Supervised classification with bimodal genes capture tissue specific and infectious disease specific signatures in microarray data

**Figure 5:**
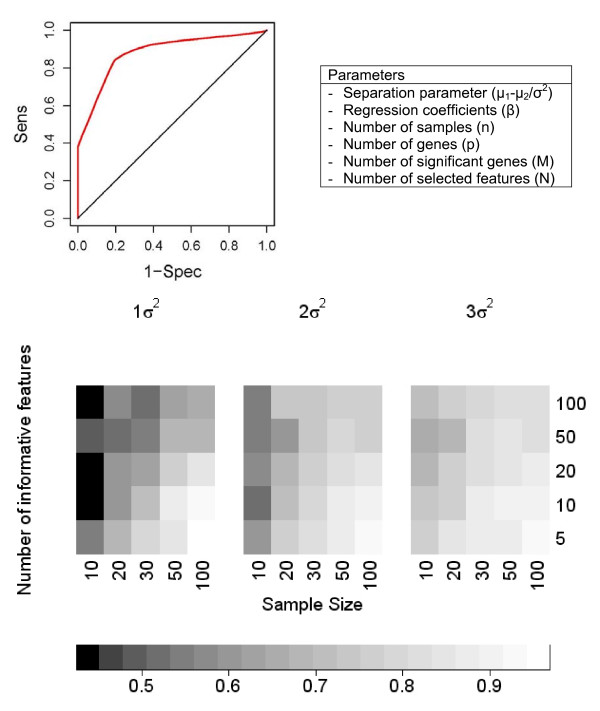
**Effect of sample size, separation and number of informative genes on classification of simulated expression data**. Classification accuracy is measured with the area under the receiver operating characteristic curve, which plots 1-specificity versus sensitivity as shown. Expression data was simulated controlling for the separation between classes, the number of samples and the number of genes related to class distinction.

**Figure 6:**
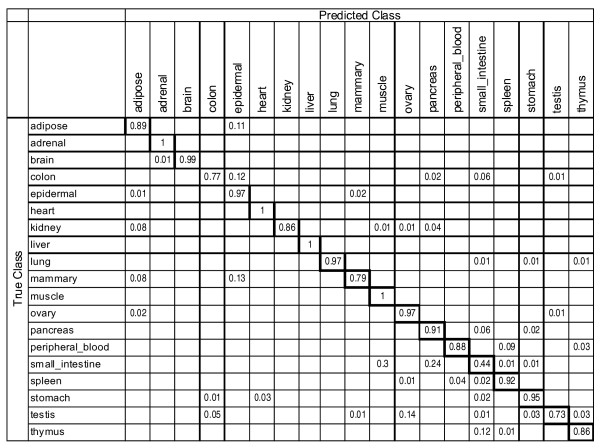
**Classification accuracy in supervised clustering of tissue phenotypes**. Values equal the proportion of true class versus predicted class membership over 100 iterations of training and testing. Values representing correct classification are outlined in bold.

**Figure 7:**
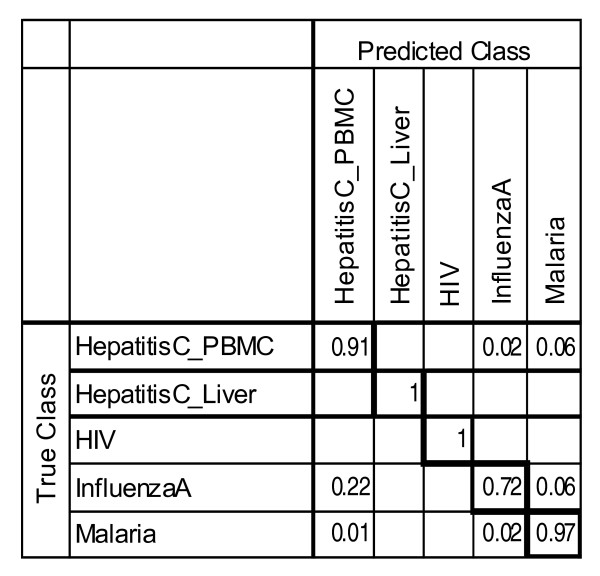
**Classification accuracy in supervised clustering of infectious disease**. Values equal the proportion of true class versus predicted class membership over 100 iterations of training and testing.

A multi-class supervised classification scheme was used to estimate whether bimodal gene expression signatures were conserved in smaller subsets of the microarray data used in our analysis of unsupervised classification and whether these signatures could be captured by a subset of just five features (Figure 5). Each dataset was split into training and test sets in a class-proportional manner such that two-thirds of the samples in each class were used for training and one-third for testing. Results over 100 independent iterations of training and testing with 5 most discriminative switch-like genes are shown in Figures 6 and 7, respectively, for tissue-specific separation and infectious disease classification. Prediction of tissue-specificity was accurate in 85% of test samples for all tissues except colon (10 samples), mammary (15 samples), small intestine (7 samples) and testis (38 samples). Microarray samples from small intestine tissue were predicted to be either muscle tissue or pancreatic tissue in 30% and 24% of test samples respectively, suggesting the persistence of cell-type-specific expression signatures in heterogeneous tissue samples. Notably, 14% of testis samples were misclassified as ovary, indicating a subset of bimodal genes may be similarly expressed in reproductive organs of the male and female. In the case of infectious diseases, multi-class supervised classification separated microarray samples from HIV-1 infection, hepatitis C and malaria well but it has allocated 22% of the influenza microarray samples to the bin for hepatitis C (Figure 6). These results indicate that tissue-specific and disease-specific bimodal gene expression profile signatures are largely conserved in independent data and can be captured with as few as five features.

We used simulated microarray data in order to gain insights on which parameters of supervised classification are determinant of the classification accuracy in datasets considered in this study. Supervised classification of simulated gene expression profiles illustrated the strong dependence of prediction accuracy on sample size, extent of separation between bimodal peaks and the number of informative genes. Classification accuracy generally improved as expression profiles became more bimodal. Increased sample size and decreased number of informative genes also resulted in more accurate classification.

## Discussion

Development and subsequent commercialization of microarray platforms has led to extensive investigation of global gene expression profiles in health and disease. Expression profiling of diverse healthy tissues provides a comprehensive perspective of the range of transcriptional regulation under physiologic conditions [42-44]. Similarly, identification of gene expression signatures indicative of disease subtypes improves our understanding of the molecular basis of pathology [7,8,45]. Small sample size and the large number of measurements for each sample are among the limiting factors that hinder the effectiveness of gene expression profiling and drive the development of new analytical methods.

Unsupervised clustering of microarray data classifies samples in an unbiased manner according to similarity in gene expression profiles. Adaptation of model-based clustering to low sample size, high dimensional datasets [23] and formalization of statistical approaches for selecting the optimum number of clusters [46] represent significant advances. In this study, we used these advanced methods to cluster and classify infectious disease and tissue phenotypes in large scale microarray data using a reduced set of 1265 switch-like genes [6,47]. Switch-like genes are identified through the detection of bimodal gene expression patterns across diverse biological conditions. Switch-like genes are likely to be under strict transcriptional regulation and are statistically enriched for cell membrane and extracellular proteins [47].

We demonstrated that model-based clustering of switch-like gene expression patterns differentiates between tissue phenotypes in a microarray dataset with tissue-specific sample sizes ranging from 5 to nearly 100. Because model-based clustering operates on the assumption that samples are drawn from multivariate Gaussian distributions, the method is particularly well-suited for the analysis of bimodal gene expression profiles. Distance-based unsupervised classification methods such as Kmeans and hierarchical clustering also led to accurate classification for tissues with large sample sizes (> 25) but had little differentiation potential at small sample sizes. The decrease in classification accuracy observed with the use of distance-based clustering may be due to estimation of the number of clusters via the gap statistic [46]. Incorporating optimization of the number of clusters into the model fitting process likely improves the performance of model-based clustering [48,49] such that tissue types with smaller sample sizes are resolved into separate clusters.

A set of 300 bimodal genes expressed on the extracellular matrix or the plasma membrane is sufficient to accurately differentiate between nineteen different tissue types in model-based clustering even at 5 microarray samples for tissue type. This set of genes includes those that code for membrane-bound integrin proteins and ECM proteins belonging to collagen, laminin, and fibronectin families. Genes expressed in the "on" mode in brain tissue and the "off" mode in muscle tissue largely coded for neural-specific cell adhesion molecules. Supervised classification has the potential to further reduce the set of 300 bimodal genes to biomarker sets when considering biomarkers for tissue-specific diseases. Accurate classification with the subset of bimodal genes presented in this article demonstrate the importance of cell/ECM interactions in tissue differentiation [25] and will prove useful as *a priori *knowledge in the analysis of microarray data produced by different laboratories.

Our study showed that the bimodal gene set identified using microarray data associated with healthy tissue is highly effective in differentiating between microarray data from tissues infected by various infectious diseases such as the HIV-1 infection, hepatitis C, influenza and malaria. The classification was unsupervised and the disease signature was conserved across laboratories. Moreover, bimodal gene sets differentiated between liver and blood cell tissues infected with the same hepatitis virus. The identification of bimodal genes expressed in the activated state in various infectious diseases and subsequent enrichment analysis with KEGG pathways provide biological context to the perturbation of various cell signaling networks induced by invading viruses. In the infectious disease states investigated here, bimodal genes expressed in the "on" mode were related to both innate and antigen-mediated immune responses.

It should be noted that other gene sets determined by feature selection may be even more discriminative of the phenotypes included in this analysis than the switch genes under consideration. Our intent in this study was not to identify discriminative genes but rather to use unsupervised clustering to determine whether switch-like expression patterns are associated with phenotype and whether previously identified switch-like genes could be used *a priori *to reduce the feature space in microarray analysis. The large body of evidence presented in this work points to the success of switch-like gene sets in capturing biologically-relevant gene expression signatures from microarray data.

Given the demonstrated biological relevance of bimodal expression patterns, it would be worthwhile to determine the clinical relevance of switch-like gene annotation. Identification of bimodal genes expressed in the activated state in complex diseases such as autism, diabetes and cancer may provide a method for dimension reduction in the identification of disease-related single nucleotide polymorphisms (SNPs) [50] and expression quantitative trait loci (eQTL) [51,52] in genome-wide association studies. Both gene sequences and promoter regions of bimodal genes expressed in the high mode identified from large scale microarray data could be searched for SNPs and eQTL linked to the onset of disease or disease progression. Further studies are needed to investigate the full potential of clinically relevant classification using switch-like gene annotation from microarray data.

## Conclusion

In this study, we showed that *a priori *knowledge gained from compilation of large-scale microarray datasets from multiple laboratories containing at least 400 samples for each gene in the array could be successfully used in reducing the dimension of features in microarray analysis. We reduced dimensionality by focusing on a set of genes with bimodal expression patterns, i.e. genes that adopt either an "on" or "off" mode of expression and are tightly regulated at the transcript level. Detection of bimodality using expectation maximization revealed a list of 1265 bimodal genes in the human genome. A subset of 300 bimodal genes was sufficient to differentiate between nineteen different tissue signatures even in small sample sizes. These genes code for proteins either on the cell membrane or at the extracellular matrix. Such proteins can be identified in tissue using fluorescence, Q dots and other methods and as such are candidate biomarkers for specific tissues.

The set of bimodal genes are capable of capturing infectious disease signatures from microarray data corresponding to hepatitis C, influenza, HIV-1 infection and malaria. Disease-specific expression patterns of bimodal genes suggest that infection by different pathogens may initiate different host responses mediated by switch-like gene expression. Determination of "on" and "off" states of switch-like genes in various tissues and diseases allowed for the identification of activated/deactivated pathways that are consistent with existing research data. Classification accuracy was exceptional even with class-specific sample sizes between ten and twenty arrays. The use of a priori knowledge from public microarray datasets in the form of bimodal gene sets has clinical implications in disease subtype classification. Genome-wide association studies for SNP discovery linked to complex diseases such as autism and cancer could potentially benefit from dimension reduction by focusing on regions of DNA that code for switch-like genes and their promoter regions.

## Methods

### Datasets

Microarray datasets used in this study were compiled from the online public repositories Gene Expression Omnibus (GEO) [53] and Array Express (AE) [54] as described in additional file2. All datasets were profiled on the HGU133A or its recently expanded version, the HGU133plus2 Affymetrix platforms. The datasets used in the study are shown in Table 1. Accession numbers of arrays used in this study are listed in Additional File 3 with corresponding phenotype information.

### Normalization

Datasets were first filtered such that only the 22,277 probe sets common to both the HGU133A and HGU133plus2 platforms were retained. Reference robust multi-chip averaging (refRMA) [55] was used for normalization. RefRMA is an adaptation of the classic RMA approach [56] that is better suited for large datasets. RMA background adjustment was applied to each array and then the arrays were normalized by fitting probe level intensities for each chip to an empirical distribution obtained by applying quantile normalization to an 800-array training set [47]. Probe affinity effects were estimated by median polishing on the training set and used to adjust the normalized probe level measures. Following these steps, probe set expression values were derived from the median value of constituent probe level intensities.

### Probe set annotation

Probe sets were annotated using Entrez Gene ID, Ensembl accession number, gene symbol, Gene Ontology terms [57] and KEGG pathways [58]. Gene identifiers and gene ontology terms were obtained from the HGU133plus2 annotation information on the Affymetrix website in March 2008. KEGG pathway annotations were obtained from the KEGG ftp site on April 28th, 2008.

### Identification of bimodal genes

Bimodal genes were identified in expression data of healthy tissues using a statistical method previously applied in the detection of switch-like behavior among mouse [47] and human [6] genes. The expectation maximization method thus employed has also been used to detect bimodality in blood glucose concentrations [59,60]. For each gene, we tested the hypothesis that the expression distribution fits a two-component Gaussian mixture model versus the null hypothesis that expression follows a single normal distribution. To correct for skewness observed in expression profiles, we used the box-cox transformation [61] as described in detail in our previous work [6,47]. The distribution of box-cox parameters over all genes was centered at zero and approximately normally distributed, suggesting that the degree of skewness is small for a majority of genes. Parameters of the two-component mixture model were fit using expectation maximization [62]. Parameters of the single normal distribution were estimated from gene-specific sample means and standard deviations. The modified log-likelihood ratio test statistic -2log*λ *was used to reject the null hypothesis. As in our previous work [6,47], p-values were generated by evaluating the chi-square distribution with six degrees of freedom at the values of the test statistic. Genes with p-values less than 0.001 were selected as candidate bimodal genes. This subset of switch-like genes was further reduced by restricting the standardized area of intersection between the distributions of the component Gaussians to 10 percent [47]. This reduction assured bimodality with significant distance between the two peaks, resulting in a list of 1265 bimodal genes. A subset of 300 bimodal genes was obtained by identifying genes with either plasma membrane and/or extracellular membrane among their cell compartment GO categories.

### Identification of "on" genes in brain, skeletal muscle, cardiac muscle, lung and infectious disease phenotypes

Bimodal gene expression values were binarized by defining a gene-specific threshold at the intersection of the probability density functions of the two-component mixture models [47]. Expression values above this threshold are described as "high" or "on". Bimodal genes in the "on" state in a majority of samples of a given phenotype were identified using a Bernoulli process [47]. Each observation or sample was modeled as an independent trial. Success was defined as expression in the "on" mode. P-values were calculated from the binomial distribution with an equal probability of success and failure. A value of p < = 0.01 indicates a significant association between bimodal gene expression and phenotype.

### Functional Enrichment

Gene sets characterized by KEGG pathways and GO terms were analyzed to identify functional categories enriched in sets of bimodal genes biased to the "on" or "off" mode in healthy and disease phenotypes. We assessed the enrichment of functional gene sets by comparing the number of "on" or "off" genes observed in a particular functional group to the number expected by chance [63]. The hypergeometric test was used to assign significance to the enriched functional gene sets. In functional enrichment, p-values less than 0.001 were considered significant.

### Distance-based clustering

Two distance-based clustering algorithms, Kmeans [64] and hierarchical clustering [9], were implemented in the R statistical environment in order to classify tissue samples into groups with similar expressions of bimodal genes. In both cases, we used Euclidean distance as the distance metric. In our implementation of Kmeans, we ran ten iterations with different initial cluster centroid locations and retained the cluster partition associated with the minimal within-cluster sum of squares. In hierarchical clustering, we used complete linkage to define the distance between clusters and observations [65]. A single cluster solution was obtained from the resulting dendrogram by cutting the tree at a level which produced the desired number of clusters. In both of these algorithms, the data-driven optimal number of clusters was determined using the gap statistic, as described below.

### Definition of the number of clusters in distance-based clustering

The optimal number of clusters $\hat{K}$ in distance-based clustering was determined with the use of the gap statistic [46]. The gap statistic tests the null hypothesis that $\hat{K}$ = 1 i.e. no clusters. Towards this goal, we compared the within-cluster sum of squares to its expected value under the reference null distribution, generated from a uniform distribution aligned with the principal components of the data [46]. Expression data was clustered into *k *groups (*k *= 1, 2,... 25) using either Kmeans or hierarchical clustering as described above. A set of *B *reference datasets were generated by drawing samples from the reference distribution and clustered in the same manner. The gap statistic (*Gap*_*k*_) was calculated as:

(1) $$Gap_{k}=\left(1/B\right)\sum_{b}\log\left(W_{kb}^{*}\right)-\log\left(W_{k}\right)$$

in which W_kb _*, (*b *= 1, 2,... *B *and *k *= 1, 2,... 25) and W_*k *_are within-cluster sums of squares of the reference and observed datasets respectively. The estimated number of clusters $\hat{K}$ is the smallest value *k *at which:

(2) *Gap*_*k *_≥ *Gap*_*k*+1 _- *s*_*k*+1 _

(3) $$s_{k}=sd_{k}\sqrt{\left(1+1/B\right)}$$

and sd_*k *_is the standard deviation of log(W_kb_*).

### Model-based subspace clustering

A model-based clustering algorithm [23], developed for the analysis of comparative genomic hybridization data, was used to cluster tissue samples on the basis of bimodal gene expression. In this approach, clusters are identified by finding an optimal partition of samples into K groups defined by cluster-specific multivariate Gaussian distributions. It is assumed that clusters can be differentiated by shifts in the mean expression values for a subset of genes and samples. Each sample is modeled as follows:

(4) *y*_*i *_= *μ *+ *r*_*i *_× *δ*_*i *_+ *ε*_*i*_

in which *y*_*i *_is the expression value in sample *i*, *μ *is a vector of mean expression values over all samples, *r*_*i *_∈ (0,1)^m ^indicates the relevant genes, *δ*_*i *_is a vector of mean shifts and *ε*_*i *_is a vector of the variance in expression values. Cluster-specific parameters Θ = (*r*_*i*_, *δi*) are sampled from a baseline distribution f_0 _in a Polya urn scheme or Chinese restaurant process as described by Hoff:

(5) $$\begin{array}{l} \text{sample }\Theta_{1}\sim {\text{f}}_{0}\\ \text{sample }\Theta_{\text{n}}\sim \alpha /(\alpha +\text{n}-1){\text{f}}_{0}+(\text{n}-1)/(\alpha +\text{n}-1){\text{f}}_{\text{n}-1} \end{array}$$

where f_n-1 _is the empirical distribution of Θ_1_,..., Θn and *α *is a constant. This process potentially results in less than n unique draws from the baseline distribution and therefore naturally leads to clustering. Parameters of the model are fit from the data using a Gibbs sampling algorithm [23]. We ran the model-based clustering algorithm [23] in the R statistical environment on 25 parallel Markov chains with 250 iterations each. We found that each chain quickly converged to equally likely, unique solutions, indicating a multi-modal posterior distribution. To obtain an approximation of the true posterior distribution, we took the average of the cluster partition with the highest log-likelihood from each chain as reported elsewhere [20,21].

### Pairwise posterior probabilities

Given a set of clusters obtained from Gibbs sampling, the probability that two observations belong to the same class is approximated by the proportion of clusters in which they are grouped together [66]. For each pair of samples, the pairwise posterior probability matrix was calculated as:

(6) $$P_{ij}=\frac{\#of\;clusters\;in\;which\;c_{i}=c_{j}}{total\#of\;clusters}$$

in which *c*_*i *_(*i *= 1,..., n samples) is a vector indicating which cluster sample *i *is assigned to. Although the pairwise posterior probability is a useful measure in itself, it does not provide a single cluster partition. For this purpose, a distance metric (*D*_*ij*_) was defined from the pairwise posterior probabilities equal to *D*_*ij *_= 1 - *P*_*ij *_[64]. A unique cluster partition can then be found using the complete linkage method, such that cluster objects are maximally separated between clusters.

### Quantifying the agreement between observed clusters and known phenotype

In this study, clustering algorithms were applied to data in which the true class membership of all samples was known *a priori*. The Adjusted Rand Index (ARI) was used to measure the amount of agreement between the known and estimated class membership [19,22]. Given two partitions of n observations U = (u1,..., uR) and V = (v1,..., vC), where U indicates the cluster partition and V indicates the true class, the Adjusted Rand Index can be calculated from the contingency table of the two partitions (Table 5). An element n_ij _of the contingency table equals the number of observations in cluster i of class j. Row sums of the contingency table are equal to n_i. _and column sums are equal to n_.j_. With this notation, the Adjusted Rand Index is calculated by the formula below and takes a value of 1 when the two partitions agree completely and a value of 0 when the index equals its expected value i.e. the partitions are no better than random.

**Table 5:** Contingency table comparing two partitions

	***v***_1_	***v***_2_	⋯	***v***_*C*_	
***u***_1_	*N*_11_	*N*_12_	⋯	*n*_1*C*_	*n*_1._

***u***_2_	*N*_21_	*N*_22_	⋯	*n*_2*C*_	*n*_2._

⋯	⋯	⋯		⋯	⋯

***u***_*R*_	*n*_*R*1_	*n*_*R*2_	⋯	*n*_*RC*_	*n*_*R*._

	*n*_.1_	*n*_.2_	⋯	*n*_.*C*_	*n*_.. _= *n*

(7) $$ARI=\frac{\sum_{i,j}\left(\begin{array}{c} n_{ij}\\ 2 \end{array}\right)-\left[\sum_{i}\left(\begin{array}{c} n_{i.}\\ 2 \end{array}\right)\sum_{j}\left(\begin{array}{c} n_{.j}\\ 2 \end{array}\right)\right]/\left(\begin{array}{c} n\\ 2 \end{array}\right)}{\frac{1}{2}\left[\sum_{i}\left(\begin{array}{c} n_{i.}\\ 2 \end{array}\right)+\sum_{j}\left(\begin{array}{c} n_{.j}\\ 2 \end{array}\right)\right]-\left[\sum_{i}\left(\begin{array}{c} n_{i.}\\ 2 \end{array}\right)\sum_{j}\left(\begin{array}{c} n_{.j}\\ 2 \end{array}\right)\right]/2}$$

### Supervised Classification

A multi-class supervised learning scheme was used to classify tissue samples on the basis of bimodal gene expression. In binary classification of microarray data, training data was used to rank features by a two-class test statistic [67]. Discriminative genes were selected from the top of this ranked list. A decision rule associated with class distinction in the set of training samples was defined on the basis of the expression of the selected genes. The decision rule was then evaluated on an independent set of samples. To extend the supervised learning scheme to multiple class problems, we trained separate classifiers to identify tissue samples of each class vs. all others [68]. Results are based on 100 independent iterations of the following training and testing procedure. Prior to classification, datasets were divided into training and testing sets in a class-proportional manner such that two-thirds of the samples in each class were used for training and one-third for testing. For the *j*th classifier (*j *= 1,..., number of classes), training samples in class *j *were assigned to class 1. All other samples were assigned to class 0. Discriminative bimodal genes were identified from the training data according to the ratio of within class to between class sums of squares [67]. Diagonal linear discriminant analysis was used to define the distances between test sample *i *and samples in class 0 (*d*_*co*_) and class 1 (*d*_*c*1_), respectively [67]. A confidence measure, defined from 0 to 1, was calculated as *d*_*co*_/(*d*_*co*_+*d*_*c*1_). Values close to 0/1 indicate low/high confidence that test sample *i *belongs to class *j*. Confidence measures were compared from each classifier and test sample *i *was assigned to the class associated with the highest confidence.

### Simulated Data

Synthetic data was used to determine the effect of sample size, effect size and the number of informative genes on prediction accuracy in binary classification. *In silico *expression datasets consisted of 10, 20, 30, 50, or 100 observations/arrays and 1000 features/genes. Initially, a binary vector indicating the class membership of each observation was drawn from a binomial distribution B(n,0.5). A number of 5, 10, 20, 50, or 100 informative gene expression profiles were drawn from a pair of multivariate normal distributions N_1_(*μ*_1_, Σ) and N_2_(*μ*_2_, Σ) representing each class of observations. Non-informative expression values representing noise genes were drawn from a mixture of N_1 _and N_2 _with mixing probabilities of 1/2 from each distribution. A diagonal covariance matrix (Σ) was used to simulate independent expression values. Effect size was measured by a separation parameter defined for each gene, specifically the distance in class-specific means divided by the pooled variance. Three effect sizes (6, 2, 1) were investigated. We used logistic regression, implemented in the stats package in the R statistical environment, to generate the response variable that indicates class membership from the expression data. Regression coefficients associated with the informative genes were drawn from a uniform distribution U(0.1,1). By logistic regression, the probability that the *i*th observation is class 1 is given by *π*_*i*_:

(8) $$\pi_{i}=\frac{1}{1+\exp\left(\beta_{1}x_{1,i}+\ldots +\beta_{M}x_{M,i}\right)}$$

in which *β*_1 _... *β*_*M *_are the defined regression coefficients and x_1, *i *_... x_*M*, *i *_are the expression values of the informative genes in the *i*th observation [69]. The simulated dataset was completed by drawing the response variable y_*i *_on the basis of *π*_*i *_(y_*i *_= 1 iff *π*_*i *_> 0.5). In this manner, the relationship between the *j*th gene and the response variable y_*i *_can be specified exactly (i.e. the value of *β*), independent of the sample distribution of gene *j*.

## Abbreviations

ARI: Adjusted Rand Index; GO: Gene Ontology; KEGG: Kyoto Encyclopedia of Genes and Genomes; ECM: extracellular matrix; MEM: membrane; PBMC: peripheral blood mononuclear cells; HIV: human immunodeficiency virus; refRMA: reference robust multi-array average; SNP: single nucleotide polymorphism; eQTL: expression quantitative trait loci; AUC: area under the receiver operating characteristic curve.

## Authors' contributions

MG and AT conceived and developed the research plan and wrote the manuscript draft. MG implemented classification of expression data and all subsequent analysis. Both authors read and approved the final manuscript.

## Supplementary Material

Additional file 1 **Annotation information for 1265 bimodal genes**. For each gene, the Affymetrix probe set ID, full gene name, and mode of expression (1 = "on", 0 = "off") in brain, skeletal muscle, cardiac muscle and lung tissue are listed. Click here for file

Additional file 3 **gormley tozeren bmc bioinformatics.** Click here for file

Additional file 2 **Annotation information for microarrays**. For each gene expression array analyzed, the GEO series and sample accession numbers are listed along with phenotype and chip type. Click here for file

## References

[B1] Arora A, Simpson DA (2008). Individual mRNA expression profiles reveal the effects of specific microRNAs. Genome biology.

[B2] Hobert O (2008). Gene regulation by transcription factors and microRNAs. Science (New York, NY).

[B3] Jaenisch R, Bird A (2003). Epigenetic regulation of gene expression: how the genome integrates intrinsic and environmental signals. Nature genetics.

[B4] Hsiao LL, Dangond F, Yoshida T, Hong R, Jensen RV, Misra J, Dillon W, Lee KF, Clark KE, Haverty P (2001). A compendium of gene expression in normal human tissues. Physiological genomics.

[B5] Warrington JA, Nair A, Mahadevappa M, Tsyganskaya M (2000). Comparison of human adult and fetal expression and identification of 535 housekeeping/maintenance genes. Physiological genomics.

[B6] Ertel A, Tozeren A (2008). Human switch-like genes and their regulation via transcription initiation and histone methylation. BMC Genomics.

[B7] Alizadeh AA, Eisen MB, Davis RE, Ma C, Lossos IS, Rosenwald A, Boldrick JC, Sabet H, Tran T, Yu X (2000). Distinct types of diffuse large B-cell lymphoma identified by gene expression profiling. Nature.

[B8] Alon U, Barkai N, Notterman DA, Gish K, Ybarra S, Mack D, Levine AJ (1999). Broad patterns of gene expression revealed by clustering analysis of tumor and normal colon tissues probed by oligonucleotide arrays. Proceedings of the National Academy of Sciences of the United States of America.

[B9] Eisen MB, Spellman PT, Brown PO, Botstein D (1998). Cluster analysis and display of genome-wide expression patterns. Proceedings of the National Academy of Sciences of the United States of America.

[B10] Subramanian A, Tamayo P, Mootha VK, Mukherjee S, Ebert BL, Gillette MA, Paulovich A, Pomeroy SL, Golub TR, Lander ES (2005). Gene set enrichment analysis: a knowledge-based approach for interpreting genome-wide expression profiles. Proceedings of the National Academy of Sciences of the United States of America.

[B11] Tusher VG, Tibshirani R, Chu G (2001). Significance analysis of microarrays applied to the ionizing radiation response. Proceedings of the National Academy of Sciences of the United States of America.

[B12] van't Veer LJ, Dai H, Vijver MJ van de, He YD, Hart AA, Mao M, Peterse HL, Kooy K van der, Marton MJ, Witteveen AT (2002). Gene expression profiling predicts clinical outcome of breast cancer. Nature.

[B13] Wang Y, Klijn JG, Zhang Y, Sieuwerts AM, Look MP, Yang F, Talantov D, Timmermans M, Meijer-van Gelder ME, Yu J (2005). Gene-expression profiles to predict distant metastasis of lymph-node-negative primary breast cancer. Lancet.

[B14] West M, Blanchette C, Dressman H, Huang E, Ishida S, Spang R, Zuzan H, Olson JA, Marks JR, Nevins JR (2001). Predicting the clinical status of human breast cancer by using gene expression profiles. Proceedings of the National Academy of Sciences of the United States of America.

[B15] Tavazoie S, Hughes JD, Campbell MJ, Cho RJ, Church GM (1999). Systematic determination of genetic network architecture. Nature genetics.

[B16] Toronen P, Kolehmainen M, Wong G, Castren E (1999). Analysis of gene expression data using self-organizing maps. FEBS letters.

[B17] Ghosh D, Chinnaiyan AM (2002). Mixture modelling of gene expression data from microarray experiments. Bioinformatics (Oxford, England).

[B18] McLachlan GJ, Bean RW, Peel D (2002). A mixture model-based approach to the clustering of microarray expression data. Bioinformatics (Oxford, England).

[B19] Yeung KY, Fraley C, Murua A, Raftery AE, Ruzzo WL (2001). Model-based clustering and data transformations for gene expression data. Bioinformatics (Oxford, England).

[B20] Joshi A, Peer Y Van de, Michoel T (2008). Analysis of a Gibbs sampler method for model-based clustering of gene expression data. Bioinformatics (Oxford, England).

[B21] Medvedovic M, Yeung KY, Bumgarner RE (2004). Bayesian mixture model based clustering of replicated microarray data. Bioinformatics (Oxford, England).

[B22] Qin ZS (2006). Clustering microarray gene expression data using weighted Chinese restaurant process. Bioinformatics (Oxford, England).

[B23] Hoff PD (2006). Model-based subspace clustering. Bayesian Analysis.

[B24] Hynes RO (2002). Integrins: bidirectional, allosteric signaling machines. Cell.

[B25] Nelson CM, Bissell MJ (2006). Of extracellular matrix, scaffolds, and signaling: tissue architecture regulates development, homeostasis, and cancer. Annual review of cell and developmental biology.

[B26] Bon G, Folgiero V, Di Carlo S, Sacchi A, Falcioni R (2007). Involvement of alpha6beta4 integrin in the mechanisms that regulate breast cancer progression. Breast Cancer Res.

[B27] Buttery RC, Rintoul RC, Sethi T (2004). Small cell lung cancer: the importance of the extracellular matrix. The international journal of biochemistry & cell biology.

[B28] van Horssen J, Dijkstra CD, de Vries HE (2007). The extracellular matrix in multiple sclerosis pathology. Journal of neurochemistry.

[B29] Yu WM, Yu H, Chen ZL (2007). Laminins in peripheral nerve development and muscular dystrophy. Molecular neurobiology.

[B30] Yamada S, Nelson WJ (2007). Synapses: sites of cell recognition, adhesion, and functional specification. Annual review of biochemistry.

[B31] Scherer SS, Arroyo EJ (2002). Recent progress on the molecular organization of myelinated axons. J Peripher Nerv Syst.

[B32] Janeway CA, Medzhitov R (2002). Innate immune recognition. Annual review of immunology.

[B33] Pasare C, Medzhitov R (2004). Toll-like receptors: linking innate and adaptive immunity. Microbes and infection/Institut Pasteur.

[B34] Bureau C, Bernad J, Chaouche N, Orfila C, Beraud M, Gonindard C, Alric L, Vinel JP, Pipy B (2001). Nonstructural 3 protein of hepatitis C virus triggers an oxidative burst in human monocytes via activation of NADPH oxidase. The Journal of biological chemistry.

[B35] Guida M, D'Elia G, Benvestito S, Casamassima A, Micelli G, Quaranta M, Moschetta R, De Lena M, Lorusso V (2002). Hepatitis C virus infection in patients with B-cell lymphoproliferative disorders. Leukemia.

[B36] Landau DA, Saadoun D, Calabrese LH, Cacoub P (2007). The pathophysiology of HCV induced B-cell clonal disorders. Autoimmunity reviews.

[B37] Lindenschmidt EG, Granato CH, Katzner K, Laufs R (1985). Evidence for limited humoral immunoglobulin M antibody response to hepatitis B core antigen during acute and chronic hepatitis B virus infections. Journal of clinical microbiology.

[B38] Sarantis H, Gray-Owen SD (2007). The specific innate immune receptor CEACAM3 triggers neutrophil bactericidal activities via a Syk kinase-dependent pathway. Cellular microbiology.

[B39] Anand AR, Ganju RK (2006). HIV-1 gp120-mediated apoptosis of T cells is regulated by the membrane tyrosine phosphatase CD45. The Journal of biological chemistry.

[B40] Barat C, Tremblay MJ (2002). Engagement of CD43 enhances human immunodeficiency virus type 1 transcriptional activity and virus production that is induced upon TCR/CD3 stimulation. The Journal of biological chemistry.

[B41] Perfettini JL, Roumier T, Castedo M, Larochette N, Boya P, Raynal B, Lazar V, Ciccosanti F, Nardacci R, Penninger J (2004). NF-kappaB and p53 are the dominant apoptosis-inducing transcription factors elicited by the HIV-1 envelope. The Journal of experimental medicine.

[B42] Shyamsundar R, Kim YH, Higgins JP, Montgomery K, Jorden M, Sethuraman A, Rijn M van de, Botstein D, Brown PO, Pollack JR (2005). A DNA microarray survey of gene expression in normal human tissues. Genome biology.

[B43] Whitney AR, Diehn M, Popper SJ, Alizadeh AA, Boldrick JC, Relman DA, Brown PO (2003). Individuality and variation in gene expression patterns in human blood. Proceedings of the National Academy of Sciences of the United States of America.

[B44] Yanai I, Benjamin H, Shmoish M, Chalifa-Caspi V, Shklar M, Ophir R, Bar-Even A, Horn-Saban S, Safran M, Domany E (2005). Genome-wide midrange transcription profiles reveal expression level relationships in human tissue specification. Bioinformatics (Oxford, England).

[B45] Golub TR, Slonim DK, Tamayo P, Huard C, Gaasenbeek M, Mesirov JP, Coller H, Loh ML, Downing JR, Caligiuri MA (1999). Molecular classification of cancer: class discovery and class prediction by gene expression monitoring. Science.

[B46] Tibshirani R, Walther G, Hastie T (2001). Estimating the number of clusters in a data set via the gap statistic. Journal of the Royal Statistical Society Series B-Statistical Methodology.

[B47] Ertel A, Tozeren A (2008). Switch-like genes populate cell communication pathways and are enriched for extracellular proteins. BMC Genomics.

[B48] Yeung KY, Medvedovic M, Bumgarner RE (2003). Clustering gene-expression data with repeated measurements. Genome Biology.

[B49] Thalamuthu A, Mukhopadhyay I, Zheng X, Tseng GC (2006). Evaluation and comparision of gene clustering methods in microarray analysis. Bioinformatics.

[B50] The Wellcome Trust Case Control Consortium (2007). Genome-wide association study of 14,000 cases of seven common diseases and 3,000 shared controls. Nature.

[B51] Stranger BE, Forrest MS, Clark AG, Minichiello MJ, Deutsch S, Lyle R, Hunt S, Kahl B, Antonarakis SE, Tavare S (2005). Genome-wide associations of gene expression variation in humans. PLoS genetics.

[B52] Stranger BE, Forrest MS, Dunning M, Ingle CE, Beazley C, Thorne N, Redon R, Bird CP, de Grassi A, Lee C (2007). Relative impact of nucleotide and copy number variation on gene expression phenotypes. Science.

[B53] Edgar R, Domrachev M, Lash AE (2002). Gene Expression Omnibus: NCBI gene expression and hybridization array data repository. Nucleic acids research.

[B54] Parkinson H, Kapushesky M, Shojatalab M, Abeygunawardena N, Coulson R, Farne A, Holloway E, Kolesnykov N, Lilja P, Lukk M (2007). ArrayExpress – a public database of microarray experiments and gene expression profiles. Nucleic acids research.

[B55] Katz S, Irizarry RA, Lin X, Tripputi M, Porter MW (2006). A summarization approach for Affymetrix GeneChip data using a reference training set from a large, biologically diverse database. BMC bioinformatics.

[B56] Irizarry RA, Hobbs B, Collin F, Beazer-Barclay YD, Antonellis KJ, Scherf U, Speed TP (2003). Exploration, normalization, and summaries of high density oligonucleotide array probe level data. Biostatistics (Oxford, England).

[B57] Ashburner M, Ball CA, Blake JA, Botstein D, Butler H, Cherry JM, Davis AP, Dolinski K, Dwight SS, Eppig JT (2000). Gene ontology: tool for the unification of biology. The Gene Ontology Consortium. Nature genetics.

[B58] Kanehisa M, Goto S (2000). KEGG: kyoto encyclopedia of genes and genomes. Nucleic acids research.

[B59] Fan J, May SJ, Zhou Y, Barrett-Connor E (2005). Bimodality of 2-h plasma glucose distributions in whites: the Rancho Bernardo study. Diabetes care.

[B60] Lim TO, Bakri R, Morad Z, Hamid MA (2002). Bimodality in blood glucose distribution: is it universal?. Diabetes care.

[B61] Maclean CJ, Morton NE, Elston RC, Yee S (1976). Skewness in commingled distributions. Biometrics.

[B62] Dempster AP, Laird NM, Rubin DB (1977). Maximum likelihood from incomplete data via the EM alogrithm. Journal of the Royal Statistical Society.

[B63] Zhang B, Kirov S, Snoddy J (2005). WebGestalt: an integrated system for exploring gene sets in various biological contexts. Nucleic acids research.

[B64] Hartigan JA, Wong MA (1979). A K-means clustering algorithm. Applied Statistics.

[B65] Gibbons FD, Roth FP (2002). Judging the quality of gene expression-based clustering methods using gene annotation. Genome Research.

[B66] Medvedovic M, Sivaganesan S (2002). Bayesian infinite mixture model based clustering of gene expression profiles. Bioinformatics (Oxford, England).

[B67] Dudoit S, Fridlyand J, Speed TP (2002). Comparison of discrimination methods for the classification of tumors using gene expression data. Journal of the American Statistical Association.

[B68] Ramaswamy S, Tamayo P, Rifkin R, Mukherjee S, Yeang CH, Angelo M, Ladd C, Reich M, Latulippe E, Mesirov JP (2001). Multiclass cancer diagnosis using tumor gene expression signatures. Proceedings of the National Academy of Sciences of the United States of America.

[B69] Venables WN, Ripley BD (2002). Modern Applied Statistics with S.

