# Supplementary material for: Expression profiles of switch-like genes accurately classify tissue and infectious disease phenotypes in model-based classification
Source: BMC Bioinformatics. 2008 Nov 17;9:486. doi: 10.1186/1471-2105-9-486 (PMC2620272; doi:10.1186/1471-2105-9-486)
Supplement: Additional file 3 — gormley tozeren bmc bioinformatics. [file 1471-2105-9-486-S3.doc]

**Expression profiles of switch-like genes accurately classify tissue and infectious**

**disease phenotypes in model-based classification**

Michael Gormley and Aydin Tozeren

Drexel University

Center for Integrated Bioinformatics

| 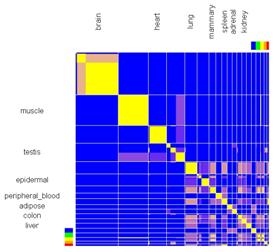 | 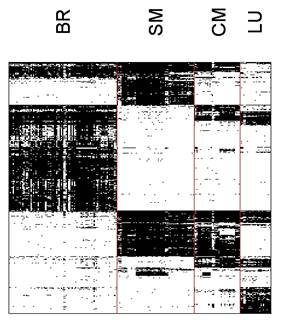 |
| --- | --- |

This page contains links to the accompanying code and necessary files for Gormley and Tozeren, "Expression profiles of switch-like genes accurately classify tissue and disease phenotypes in model-based classification".   Code is written in the R statistical environment.  Matlab is also required for box-cox transformation.  A number of R packages are used in this analysis.  These are listed below, along with directions for how to install them.

Packages: R.matlab, mvtnorm, gplots, SparseM, mclust, Biobase, sma, igraph, snow, Rmpi, Rlecuyer

All of the packages except for Biobase are available from the Comprehensive R Archive Network (CRAN).  These packages can be installed by typing the following at the R prompt:

install.packages('*package.name*')

Installation is completed by following the on-screen instructions. Biobase is available from the Bioconductor project.  To install, type the following at the R prompt:

source("http://bioconductor.org/biocLite.R")

biocLite()

The Relevant Fils link can be used to obtain the processed, normalized microarray data and necessary phenotype information from the tissue type dataset discussed in the manuscript. Two text files are also given which can lead the user through the analyses described. One file uses a parallel implementation of R to run portions of the code. This implementation can only be run on unix systems with the R package snow installed. The other batch file has no parallel implementation but will take much longer to run. These files can be run in R in batch mode to produce the output for the analyses described in the manuscript. To run R in batch mode, type the following in the command line:

R CMD BATCH *filename.txt* out.txt

If you are running these codes on a windows machine, you will need to add some files to the path in order to run R from the command line.  Go to Start>Control Panel>System>Advanced>Environmental Variables, select Path and click edit.  Add the bin directory under the R directory to your path. (ie. C:\R\R-2.7.1\bin).  Additionally, you will need to install the Rtools software.  During installation, make sure to select the option to change the path extension.

Links

- [R Project for Statistical Computing](http://www.r-project.org/)
- [Bioconductor](http://bioconductor.org/)
- [Rtools](http://www.murdoch-sutherland.com/Rtools/)
- [Relevant Files](http://bioinformatics.biomed.drexel.edu/Mike_files/Files.htm)
- [Relevant Codes](http://bioinformatics.biomed.drexel.edu/Mike_files/Codes.htm)
